# MR_NET: A Method for Breast Cancer Detection and Localization from Histological Images Through Explainable Convolutional Neural Networks

**DOI:** 10.3390/s24217022

**Published:** 2024-10-31

**Authors:** Rachele Catalano, Myriam Giusy Tibaldi, Lucia Lombardi, Antonella Santone, Mario Cesarelli, Francesco Mercaldo

**Affiliations:** 1 Department of Medicine and Health Sciences “Vincenzo Tiberio”, University of Molise, 86100 Campobasso, Italy; r.catalano1@studenti.unimol.it (R.C.); l.lombardi12@studenti.unimol.it (L.L.); antonella.santone@unimol.it (A.S.); 2 Department of Engineering, University of Sannio, 82100 Benevento, Italy; mcesarelli@unisannio.it

**Keywords:** convolutional neural networks, artificial intelligence, deep learning, digital pathology, breast cancer

## Abstract

Breast cancer is the most prevalent cancer among women globally, making early and accurate detection essential for effective treatment and improved survival rates. This paper presents a method designed to detect and localize breast cancer using deep learning, specifically convolutional neural networks. The approach classifies histological images of breast tissue as either tumor-positive or tumor-negative. We utilize several deep learning models, including a custom-built CNN, EfficientNet, ResNet50, VGG-16, VGG-19, and MobileNet. Fine-tuning was also applied to VGG-16, VGG-19, and MobileNet to enhance performance. Additionally, we introduce a novel deep learning model called MR_Net, aimed at providing a more accurate network for breast cancer detection and localization, potentially assisting clinicians in making informed decisions. This model could also accelerate the diagnostic process, enabling early detection of the disease. Furthermore, we propose a method for explainable predictions by generating heatmaps that highlight the regions within tissue images that the model focuses on when predicting a label, revealing the detection of benign, atypical, and malignant tumors. We evaluate both the quantitative and qualitative performance of MR_Net and the other models, also presenting explainable results that allow visualization of the tissue areas identified by the model as relevant to the presence of breast cancer.

## 1. Introduction

Breast cancer (BC) is currently representing the second most common cancer and the leading cause of cancer-related death among women, following lung cancer. Currently, over 280,000 women are diagnosed with BC annually in the United States, with 44,000 succumbing to the disease [1]. Despite advancements in early detection and an improved understanding of the molecular biology of BC, nearly 30% of patients with “early-stage” BC experience disease recurrence [2]. In recent years, the mortality rate has declined, primarily due to improvements in treatment and the availability of targeted therapies, with tumor down-staging through screening programs also playing a significant role [3].

BC is characterized by the uncontrolled and abnormal growth of breast tissues, resulting in the formation of a lump or tumor [4]. These breast lesions can be categorized as either benign or malignant. Benign tumors grow slowly and do not invade nearby cells, whereas malignant tumors grow rapidly and aggressively infect surrounding cells [5]. Breast cancers are generally classified as either lobular or ductal.

Multiple risk factors for breast cancer have been identified, including hormonal, dietary, and lifestyle-related factors. Hormonal or reproductive risk factors include early age at menarche, nulliparity, older age at first birth, and late menopause. Several genetic factors also confer higher risk, most notably germline mutations in BRCA1 and BRCA2. Other mutations associated with increased BC risk include CHECK2, TP53, PTEN, ATM, CDH1, NBN, PALB2, and mismatch repair genes [6].

The diagnosis of BC typically begins with initial detection through palpation and regular check-ups using mammography or ultrasound imaging. If these examinations suggest the possibility of malignant tissue growth, a breast tissue biopsy is conducted [7]. Histological analysis of biopsy samples remains the gold standard for diagnosing many types of cancer. Breast tissue biopsies allow pathologists to assess the microscopic structure and components of the tissue, distinguishing between normal tissue, non-malignant (benign), and malignant lesions, and providing a prognostic evaluation [8].

Benign lesions represent changes in the normal structures of breast parenchyma that are not directly linked to malignancy progression. Carcinomas can be classified as in situ or invasive. In situ carcinoma is confined within the mammary ductal-lobular system, while invasive carcinoma spreads beyond this structure. The tissue collected during the biopsy is usually stained with hematoxylin and eosin (HE) before visual analysis by specialists [9]. Pathologists examine the regularities in cell shapes, density, and tissue structures by analyzing a thin slice of tissue under an optical microscope to identify cancerous regions and determine the malignancy degree. However, due to the complexity and diversity of histology images, manual examination requires extensive knowledge and experience and can be time-consuming and prone to errors [10].

To address this challenge, efforts have been made to leverage Artificial Intelligence (AI) methods as new diagnostic tools for pathologists. Deep learning, in particular, aims to enhance diagnostic accuracy and minimize human error, supporting pathologists rather than replacing them, and fostering a collaborative approach for better diagnostic outcomes [11].

Starting from these considerations, in this paper, we propose a method to automatically detect the presence of BC from histological tissue images. Specifically, we propose the adoption of convolutional neural networks (CNNs) to classify histological images of breast tissue as either tumor-positive or tumor-negative. The CNNs were trained and tested using various combinations of hyperparameters to achieve optimal results. The dataset used for the experimental analysis was developed through a collaboration between the National Cancer Institute IRCCS “Fondazione G. Pascale” in Naples, the Institute for High Performance Computing and Networks (ICAR), and IBM Research in Zurich. The images were collected between 2019 and 2020 in Naples, Italy and made publicly available in 2021. A second version was released in 2022, adding 148 additional images. The dataset contains 4539 high-resolution histological images obtained using hematoxylin and eosin (HE) staining and a magnification factor of 40×. The images vary in size, ensuring greater sample heterogeneity.

Thus, the proposed method focuses on classifying HE-stained breast histology images into three categories: benign tissue, atypical lesions, and malignant tumors. Specifically, we consider the following lesion types: Normal (N), Pathological Benign (PB), Usual Ductal Hyperplasia (UDH), Flat Epithelial Atypia (FEA), Atypical Ductal Hyperplasia (ADH), Ductal Carcinoma in Situ (DCIS), and Invasive Carcinoma (IC). The obtained results demonstrate the method’s ability to accurately distinguish between the three considered levels (atypical, benign, and malignant) and outperform other state-of-the-art methods based on feature extraction.

The proposed approach has the potential to enhance computer-assisted diagnosis of BC and improve early diagnosis, contributing to the prevention of avoidable deaths.

Below, we itemize the main contributions of this manuscript:

- We propose a method to classify HE-stained breast histology images;
- We design and develop a dedicated CNN for the breast histology image classification task, i.e., MR_NET;
- We evaluate several state-of-the-art CNN architectures with the aim to show the effectiveness of the proposed CNN;
- We take into account the explainability, aimed to show which areas of the image under analysis is symptomatic of the cancerous area, with the goal to improve the trustworthiness by medical staff and patients in deep learning;
- We evaluate the proposed CNN from a quantitative (by computing performance metrics) and a qualitative point of view (by analyzing the explainability behind the model prediction);
- We consider a dataset freely available for research purposes for replication purposes.

The paper is structured as follows: Section 2 presents the proposed method; Section 3 discusses the experimental results; Section 4 reviews the state-of-the-art literature; and finally, Section 5 concludes with future research directions.

## 2. Method

This section presents the method we propose for detecting and localizing BC from tissue images. The proposed aim was to develop a model that could classify histological images as either positive or negative for BC.

Specifically, this was a multiclass classification problem, as each tissue image was assigned to one of three possible classes based on supervised learning. All the images in the training dataset were already labeled.

**Figure 1:**
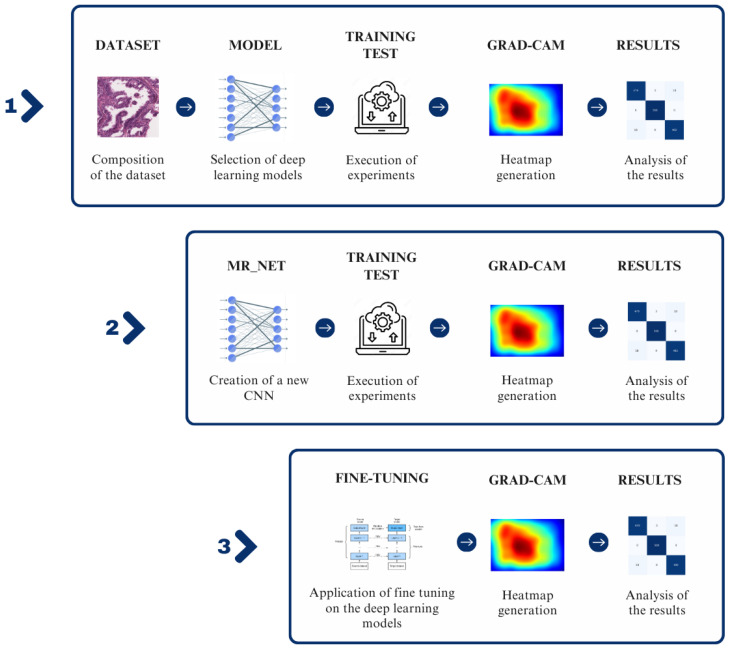
The main steps of the proposed method.

The following work was divided into three different phases, as shown in Figure 1.

- The first phase (box 1 in Figure 1) involved the choice of a dataset, selection of deep learning models, training and testing of these models, generation of explainability through Gradient-weighted Class Activation Mapping (i.e., Grad-CAM) [12,13,14,15], and an analysis of the results.
- The second phase (box 2 in Figure 1) involved the design of a new network architecture called MR_Net, execution of the experiments and generation of Grad-CAMs based on this model, and an analysis of the results.
- In the third phase (box 3 in Figure 1), fine-tuning of the previously trained networks that obtained the best performance in the first phase was carried out with consequent generation of the Grad-CAMs in order to compare the results obtained with and without fine-tuning.

### 2.1. Dataset and Preprocessing

The dataset is a critical factor in machine learning, as it directly impacts model performance, generalization, and reliability. Therefore, selecting the appropriate dataset for conducting experiments is essential. In the following case study, the BReAst Carcinoma Subtyping (BRACS) [16] dataset was adopted, consisting of histological images stained with hematoxylin and eosin. This dataset was chosen for the large number of images and the inclusion of not only normal and cancerous images, but also two atypical lesions, known as precancerous lesions. The BRACS dataset contains 6 different subtypes of lesions including also images representing atypical lesions. Moreover, histological images representing normal tissue samples are also included. The BRACS dataset is composed of hematoxylin-and-eosin-stained histopathological images. The dataset contains 547 labeled whole-slide images, 4539 Labeled regions of interest for a total of 189 patients.

In particular, the types of lesions present in this dataset are Normal (N), Pathological Benign (PB), Usual Ductal Hyperplasia (UDH), Flat Epithelial Atypia (FEA), Atypical Ductal Hyperplasia (ADH), Ductal Carcinoma in Situ (DCIS), and Invasive Carcinoma (IC).

A dataset forms the foundation for training machine learning models. The model learns patterns, features, and relationships from the examples within the dataset, so having a larger number of examples enables the model to achieve better results.

In this regard, to optimize the data, a preprocessing phase was carried out in such a way as to obtain not only a higher number of images, but also their more homogeneous distribution between the different classes. The analyzed problem was in fact based on the classification of images into three main classes: atypical, malignant, and benign.

- The atypical class included images related to Flat Epithelial Atypia (FEA) and Atypical Ductal Hyperplasia (ADH).
- The malignant class included images of Ductal Carcinoma in Situ (DCIS) and Invasive Carcinoma (IC).
- The benign class included images labeled as Normal (N), Pathological Benign (PB), and Usual Ductal Hyperplasia (UDH).

Subsequently a resizing was carried out in order to obtain a size of 500 × 500 pixels for each image to obtain images more manageable from the point of view of the disk size. To increase the number of examples to be provided to the deep learning models, data augmentation was applied, in particular the horizontal flip, brightness, and zoom techniques. The images obtained from the data augmentation process were included only in the training dataset.

Following this preprocessing phase, the final dataset used contained 5628 images of which 80% were allocated to training, 10% to testing, and another 10% to validation, obtaining the following subdivision:

- Training set: 4500 images, with 1500 classified as benign, 1500 as atypical, and 1500 as malignant.
- Validation set: 564 images, with 188 classified as benign, 188 as atypical, and 188 as malignant.
- Test set: 564 images, with 188 classified as benign, 188 as atypical, and 188 as malignant.

These considerations highlight the importance of a well-curated and diverse dataset in developing accurate and clinically relevant machine learning models for BC detection from tissue images. The dataset’s quality directly affects the model’s capacity to generalize, detect different cancer types, and ultimately contribute to the success of the diagnostic tool.

### 2.2. The CNN Models

In this paper, we considered a set of CNNs i.e., Standard_CNN, EfficientNet, ResNet50, VGG-16, VGG-19, and MobileNet. Furthermore, we present the proposed MR_NET model we designed.

**Figure 2:**
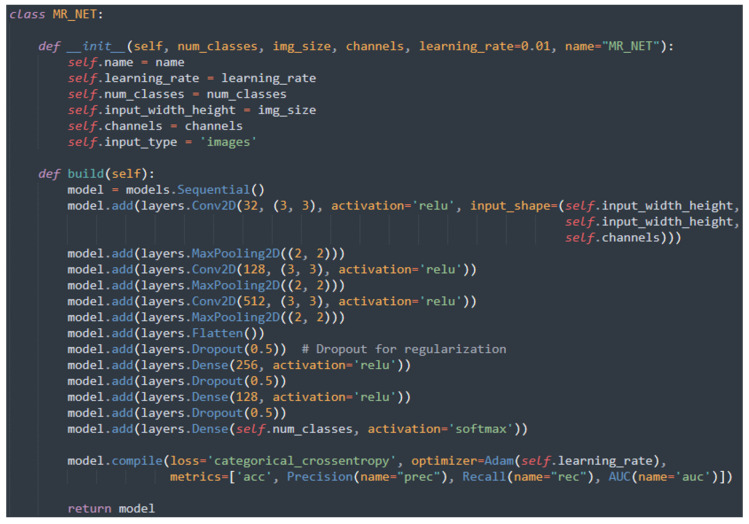
Code snippet related to the MR_Net implementation.

The following is a brief description of the CNNs we considered:

- Standard_CNN: This network consists of 13 layers. The convolutional block includes three Conv2D layers with 3 × 3 filters of sizes 32, 64, and 128, each followed by ReLU activation, and alternates with three MaxPooling2D layers. The classification block contains three Dense layers with 512 and 256 units, respectively, using ReLU activation, followed by a final layer with 3 neurons and SoftMax activation. Dropout layers with a rate of 0.5 are interspersed to regularize the network. Since it is a multiclass classification task, the network utilized the categorical cross-entropy loss function.
- EfficientNet [17]: EfficientNets are a family of CNNs built upon a concept called “compound scaling” by Mingxing Tan and Quoc V. Le [17]. They proposed a technique that used a simple compound coefficient to scale uniformly each dimension of the network (depth, width, and resolution) with a fixed ratio. They managed to develop seven models of different dimensions that achieved better accuracy and efficiency than previous ConvNets. In particular, to conduct our analysis, we resorted to the base model, EfficientNet-B0. This model consists of a total of 28 layers, divided in 9 blocks: 1 stem layer for the input, 7 fundamental blocks (whose number of layers included can vary) in the middle, and a head layer for the output. The first layer is a Conv3 × 3 that accepts input images with size 224 × 224 × 3, while the last one is a Conv1 × 1. The building blocks of this architecture are the Mobile Inverted Bottleneck (MBConv) layers, that are based on the inverted residual blocks from MobileNet, but obviously with some modifications. The MBConv layer begins with a depthwise convolution, followed by a pointwise (1 × 1) convolution that increases the number of channels. It then applies another 1 × 1 convolution to reduce the number of channels back to the original count.
- ResNet-50 [18]: ResNets, or Residual Networks, are a family of CNNs launched in 2015 by Microsoft Research Asia. ResNet-50 has this name because it consists of 50 layers: one MaxPooling layer before the main blocks, one AveragePooling at the end and 48 intermediate layers. The networks are inspired by VGG nets: the convolutional layers mostly have 3 × 3 filters, however the model has fewer filters and lower complexity. ResNets became popular because of the introduction of a new building block, the residual block, that in the case of ResNet-50 includes 3 layers, all with ReLU activation. This means that all the layers are connected; however, during the training some of the connections are “skipped” in order to preserve the information learned. The first convolutional layer takes input images with a maximum size of 224 × 224 × 3 that are then fed into the main layers. The convolutional layers are divided into 4 main blocks, each of which includes 3 layers as stated before, but repeated a different number of times. At last, before going into the classifier, the images go through an AveragePooling layer.
- VGG [19]: VGG-16 is a neural network architecture developed by the Visual Geometry Group at the University of Oxford’s engineering sciences department. The two most commonly used versions, VGG-16 and VGG-19, differ in the number of layers. Inspired by AlexNet, VGG uses smaller convolutional filters, with an architecture consisting of 5 blocks of 3 × 3 convolutional layers. The first two blocks contain 2 convolutional layers, while the last three have either 3 (in VGG-16) or 4 (in VGG-19) layers. Max-pooling layers are placed between each block, followed by a final block of 3 fully connected layers.
  The input image size is 224 × 224 × 3. In this paper, we examined two variants of this network: VGG-16 and VGG-19. The primary difference is in the number of layers—VGG-16 has 16 layers (13 convolutional and 3 fully connected), whereas VGG-19 includes 19 layers (16 convolutional and 3 fully connected). The extra layers in VGG-19 result from additional convolutional layers added in the middle of the network.
- MobileNet [20]: this network primarily employs depthwise separable convolutions instead of the standard convolutions used in earlier architectures to create more lightweight models. Each depthwise separable convolution layer is composed of a depthwise convolution followed by a pointwise convolution. When counting depthwise and pointwise convolutions as separate layers, a MobileNet contains 28 layers. The input image size is 224 × 224 × 3.
- MR_Net: The network we designed and developed in this paper is composed of 13 layers constituting a convolutional block and a classification block. The convolutional block is composed of three Conv2D layers, alternating with MaxPooling2D layers, with the aim of reducing the dimensions of the images, maintaining their main characteristics. The classification block consists of three Dense layers, alternating with Dropout layers, used to improve the generalization of the network. The ReLU` was chosen as the activation function for the intermediate layers and the Softmax for the last layer linked to the classification. Finally, since it was a multiclass classification problem, categorical cross-entropy was used for the loss of function. Figure 2 shows the code snippet related to the MR_Net Python implementation, with the aim to understand in details the network structure and layers.

### 2.3. Training

After developing the CNN models, they were trained on the selected dataset using specific hyperparameters. These included the number of epochs, batch size, and learning rate. The values that led to better results selected during the training phase are summarized in Table 1.

During the training phase, additional experiments were conducted by varying the number of epochs, learning rate, and batch size. However, all of these variations yielded worse results compared to those shown in Table 1.

In Table 1, we can see hyperparameters such as image size, epochs, batch size, and learning rate:

- The image size: this hyperparameter indicates the size of the input image which corresponds to the size of the input layer of the network. The size of the image can have different values depending on the network in which it is inserted. The typical dimensions of images for classification are 224 × 224 × 3, but in this study, we also used the dimensions 110 × 110 × 3 as seen in Table 1.
- The number of epochs is a hyperparameter that specifies how many times an algorithm processes the entire dataset. Typically, a higher number of epochs is used to enable the model to learn as much as possible. However, it is important to monitor the number of epochs closely, as an excessively high value can lead to overfitting.
- The batch size refers to the number of examples processed in each batch during training. For instance, with a batch size of 32 and a training set of 4500 examples, there would be 32 batches, each containing 140 examples. Consequently, an epoch consists of 32 iterations. It is crucial to choose an appropriate batch size, as a value that is too small (less than 10) can hinder performance optimization, while a value that is too large may lead to memory issues or increased risk of overfitting. Commonly used batch sizes are 16, 32, 64, or 128.
- The learning rate determines how frequently the neural network updates its parameters during training. If the learning rate is too high, the model might update its parameters too quickly, potentially overshooting the optimal solution. Conversely, if the learning rate is too low, updates may be too slow, which can impede convergence and necessitate more training iterations to achieve optimal results. Commonly used learning rate values are 0.01, 0.001, and 0.0001.

### 2.4. Fine-Tuning

After evaluating the performance of the state-of-the-art models and the one we designed, i.e., MR_NET (all trained from scratch), in this last phase, we considered three additional models obtained using fine-tuning to obtain better performance. Fine-tuning is a transfer learning technique involving the use of a pre-trained model, typically on a large dataset, we can adapt to a specific problem by continuing the training only for certain layers. This allows the model to adjust its weights, and especially if the dataset under analysis is small, it reduces the risk of overfitting. This condition in machine learning occurs when a neural network is too closely fit to training data, resulting in a model that is not able to generalize the representations learned and correctly apply them to make predictions on new data. Overfitting can be detected by analyzing the metrics on the output of a model, above all others, accuracy. Fine-tuning requires two main steps: feature extraction and actual fine-tuning.

- Feature extraction: it consists of using the representations learned from a model in a previous training session to extract features from new data. When dealing with pre-trained CNNs, the classifier (the last part of the network, consisting of fully connected layers) is usually discarded and only the so-called convolutional base (the first part of the network, consisting of Conv2D and pooling layers) is considered. Before a new classification task can be submitted to the model, a new classifier built specifically for the dataset must be added and then trained. While this new training occurs, the layers of the convolutional base should not update their weights, otherwise there is the chance that the representations previously learned will be lost due to the error caused by a random initialization of the weights of the classifier. This is achieved through a process called freezing, which makes all the parameters of a layer untrainable. The convolutional base is kept because the patterns it identified are more generic compared to the ones of the classifier, therefore they are also more easily applicable to various domains. A key aspect to bear in mind is that networks learn hierarchies of patterns, so that the initial layers learn local patterns, down to the last, which will recognize patterns that are gradually more global, abstract, and specific to the dataset. For this reason, if the datasets are extremely different, it is better not to freeze the entire convolutional base, but only the first few layers.
- Fine-tuning: after the classifier has been trained, some of the layers that are closer to it are unfrozen and re-trained. Training these layers together with the classifier allows the representations of these layers to be adapted to the specific dataset. Usually, rather low learning rates are used so the weights are not completely modified. The choice of the number of layers to be re-trained must be made wisely, as the more parameters are trained, the greater the risk of overfitting on a small dataset like ours.

Based on the results obtained in the first part of the analysis we conducted, we chose to consider the most promising networks, namely, MobileNet, VGG-16, and VGG-19. We created new models in which we imported the three networks with the weights obtained from training on the ImageNet dataset, respectively, and then froze the entire convolutional base so that it could not be trained. We extended the model by adding the layers of the new classifier and trained it, thus starting the feature extraction phase. Depending on the model, we then unfroze several layers before further training, as required by the second step of fine-tuning. According to the layer names adopted in Keras models, we unfroze the following:

- For MobileNet, the weights of the layers in the last two convolutional blocks, starting with “conv_dw_12”.
- For VGG-16, the weights of all three layers in the last convolutional block, starting with “block5_conv1”.
- For VGG-19, the weights of all four layers in the last convolutional block, starting with “block5_conv1”.

If not specified, the models were trained from scratch. The fine-tuned models were pre-trained with the ImageNet weights.

**Figure 3:**
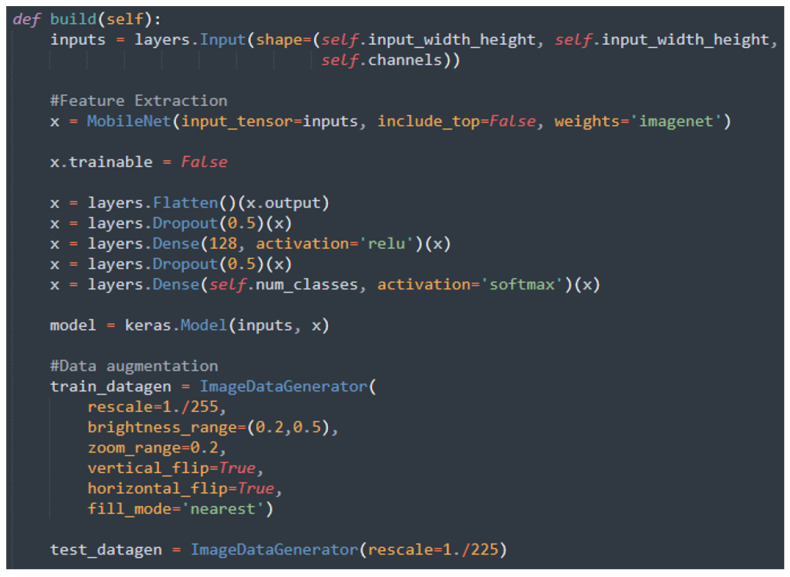
The Python code snippet related to the fine-tuning of the MobileNet network.

As an example, in Figure 3, we show the code we developed for the fine-tuning of the MobileNet network. Similar code snippets were developed for the fine-tuning of the VGG-16 and VGG-19 networks.

### 2.5. Grad-CAM

In the following, we provide details about the technique we exploited to provide explainability. Grad-CAM is a technique used in deep learning for understanding the decisions made by CNNs in image classification tasks. In a nutshell, it provides insights into which regions of an image the network is focusing on when making predictions, thereby offering interpretability to the model’s decisions.

Grad-CAM is typically exploited for interpretability; as a matter of fact, deep neural networks are often treated as black boxes due to their complex architectures. Grad-CAM provides insight into their decision-making process by highlighting which parts of an image are important for a particular prediction. Moreover, it can be useful for model debugging, i.e., it helps in understanding and debugging model errors. By visualizing the regions of an image that contribute most to a particular prediction, researchers can identify potential biases or misclassifications. Grad-CAM can also provide trust and transparency; as a matter of fact, in critical applications like healthcare or autonomous driving, it is crucial to understand why a model makes a certain decision. Grad-CAM enhances the trustworthiness and transparency of AI systems by providing interpretable explanations for their outputs.

In the following, we describe how the Grad-CAM is able to highlight the areas in an image under analysis that are symptomatic of a certain prediction:

- Forward pass: first, the input image is fed forward through the CNN to obtain the final convolutional feature maps.
- Backpropagation: during the training phase, gradients are calculated with respect to the predicted class score in the final layer of the CNN, typically using backpropagation.
- Gradient aggregation: The gradients flowing backward are utilized to assess the significance of each feature map in the final prediction. This is achieved by averaging the gradients of the target class across all spatial locations in the feature maps.
- Weighted combination: these gradients are then used to weight the feature maps, highlighting the regions in each feature map that are most relevant to the predicted class.
- Activation map generation: finally, the weighted combination of feature maps is passed through a ReLU activation to obtain the Grad-CAM activation map.

With the main Grad-CAM working mechanisms explained, we now provide the formal definition:

Let us use the following notations: $A^{k}$ is the *k*th convolutional feature map, where *k* ranges from 1 to the number of feature maps. $w_{k}^{c}$ is the weight associated with the *k*th feature map for class *c*. $\alpha_{k}^{c}$ is the importance score for the *k*th feature map in predicting class *c*. $L_{c}$ is the final output score for class *c*.

The Grad-CAM method computes the importance scores $\alpha_{k}^{c}$ for each feature map $A^{k}$ as follows:

$$\alpha_{k}^{c}=\frac{1}{Z}\sum_{i}\sum_{j}\frac{\partial L_{c}}{\partial A_{i,j}^{k}}$$

where $\frac{\partial L_{c}}{\partial A_{i,j}^{k}}$ represents the gradient of the class score $L_{c}$ with respect to the activation of the kth feature map at spatial location (i,j), and *Z* is a normalization factor.

Then, the Grad-CAM activation map $M^{c}$ for class *c* is obtained by the weighted combination of the feature maps $A^{k}$:

$$M^{c}=ReLU\left(\sum_{k}w_{k}^{c}A^{k}\right)$$

## 3. Experimental Analysis

In this section, we present the results of the experimental analysis we conducted that aimed to propose a reliable method for the detection and localization of BC.

In detail, we provide the metrics and the confusion matrices obtained in the classification phase to carry out a quantitative analysis. Afterwards, we introduce a more qualitative analysis by presenting the images resulting from the Grad-CAM technique; this allowed us to determine whether the models based their decisions on the right elements of the images or not.

The results refer to the classification performed using the images contained in the test set, already described in Section 2.1.

### 3.1. Quantitative Analysis

We computed the following metrics with the aim to evaluate the proposed method from a quantitative point of view:

- Accuracy is the proportion of correctly classified instances (both true positives and true negatives) out of all instances. It gives a general idea of how well the model is performing but can be misleading if the data are imbalanced (e.g., more negative cases than positive).
- Loss refers to how well the model’s predictions align with the actual outcomes.
- Precision is the proportion of correctly predicted positive instances (true positives) out of all instances predicted as positive (including false positives). It focuses on how accurate the positive predictions are.
- Recall is the proportion of correctly predicted positive instances out of all actual positive instances. It measures how well the model can identify true positives.

Table 2 and Table 3 show the results of the experimental analysis with the hyperparameters given in Table 1. Table 4 shows the results of the experimental analysis carried out with fine-tuning.

The metrics allowed us to determine which model achieved better results. We had to ensure that the values related to accuracy, precision, and recall are close to one, while the loss value should be as close to zero as possible. Considering Table 2, none of the models appeared to have achieved such low values for the loss; however, the accuracy showed promising values. The networks that obtained the best results were MobileNet and VGG-19 with an accuracy of 73%, a very satisfactory result considering that other studies conducted on the same dataset achieved an accuracy of 56% [21] and 66% [22]. However, we must underline the fact that our network was built to implement a three-class classification, while the aforementioned works attempted a seven-class classification.

As highlighted in the introduction, the main aim of this study was to find a CNN that achieved better results than Standard_CNN, with which an accuracy of 67% was obtained, a result that still remains high if compared with the state of the art.

The network we designed and developed for this purpose, MR_NET, whose structure is described in Section 2.2 obtained an accuracy percentage higher than that of the Standard_CNN, namely, 69%, as shown in Table 3. Furthermore, another striking detail is the fact that our network exhibited a lower loss value than the other models in Table 2.

The last models considered were the ones obtained through fine-tuning; their results are in Table 4. We chose to use this technique only for the top three models, namely, MobileNet, VGG-16, and VGG-19. In this analysis, we observed the accuracy achieved with this method was lower than that achieved before. In particular, the VGG-19 network only reached 71% against the 73% obtained in the previous phase.

In addition to the metrics in Table 2, it is crucial to consider the confusion matrix to see the classification quality of the proposed networks.

A confusion matrix is a representation used in machine learning and classification tasks to assess the performance of a classification algorithm. It summarizes the predicted and actual classes for a set of instances, offering insights into the algorithm’s accuracy and error patterns. This matrix is especially valuable for evaluating both binary and multiclass classification problems.

In the confusion matrix, the term “positive” means affected by an atypical or malignant tumor, and “negative” means benign.

The confusion matrices of MobileNet, VGG-19, Standard_CNN, and MR_Net are presented below.

**Figure 4:**
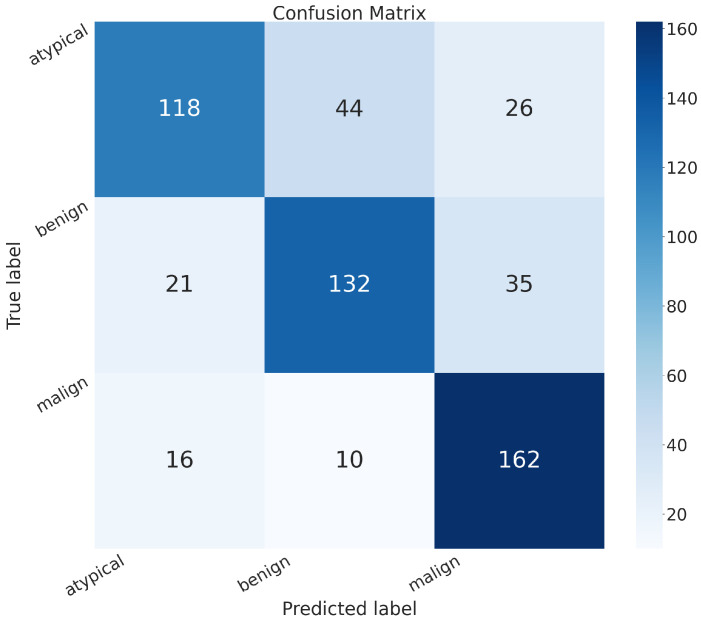
Confusion matrix obtained with the MobileNet model.

Concerning the confusion matrix shown in Figure 4, the MobileNet model shows the following:

- True positive (*TP*): 322 (patients truly positive), with 144 affected by atypical and 178 by malignant tumors.
- True negative (*TN*): 132 (patients truly negative).
- False positive (*FP*): 56 (patients negative but classified as positive).
- False negative (*FN*): 54 (patients positive but classified as negative).

**Figure 5:**
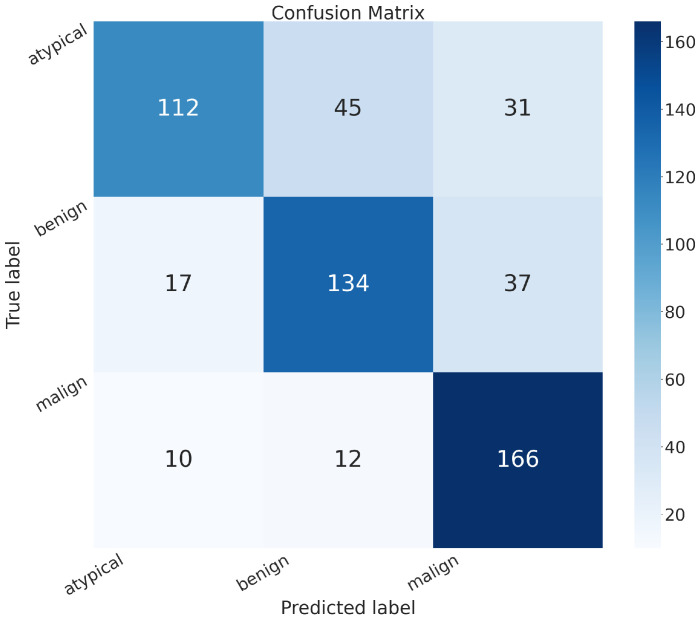
Confusion matrix obtained with the VGG-19 model.

The confusion matrix of the VGG-19 model, in Figure 5, shows the following:

- True positive (*TP*): 319 (patients truly positive), with 122 affected by atypical 197 and by malignant tumors.
- True negative (*TN*): 134 (patients truly negative).
- False positive (*FP*): 54 (patients negative but classified as positive).
- False negative (*FN*): 57 (patients positive but classified as negative).

**Figure 6:**
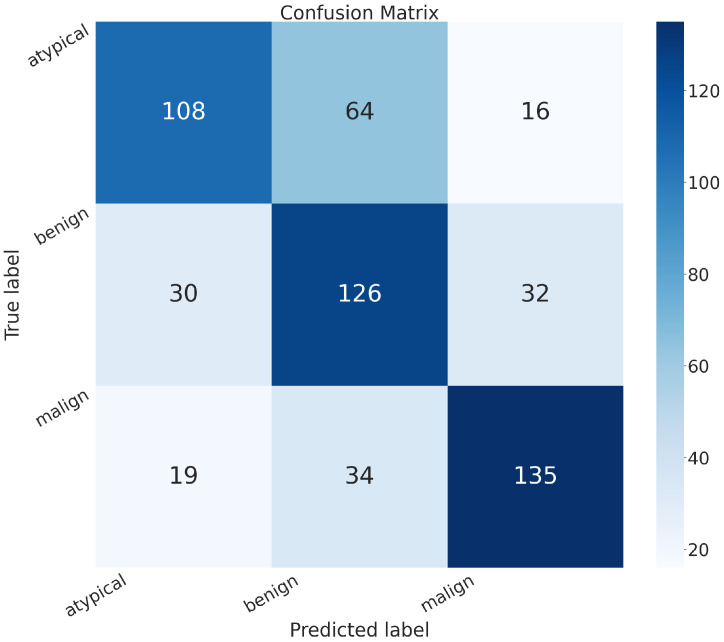
Confusion matrix obtained with the Standard_CNN model.

Figure 6 shows the confusion matrix obtained using the Standard_CNN model:

- True positive (*TP*): 278 (patients truly positive), with 127 affected by atypical and 151 by malignant tumors.
- True negative (*TN*): 126 (patients truly negative).
- False positive (*FP*): 62 (patients negative but classified as positive).
- False negative (*FN*): 98 (patients positive but classified as negative).

**Figure 7:**
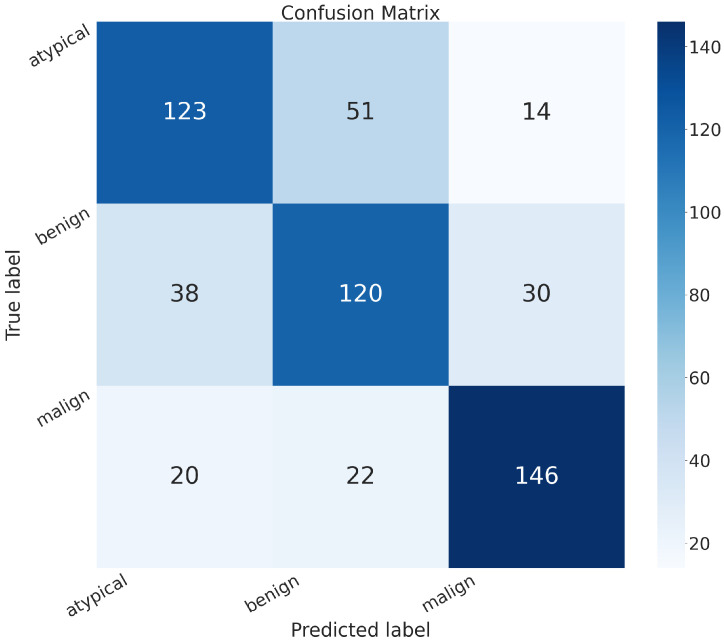
Confusion matrix obtained with the MR_NET model.

Figure 7 shows the confusion matrix related to the model we built, MR_Net:

- True positive (*TP*): 303 (patients truly positive), with 143 affected by atypical and 160 by malignant tumors.
- True negative (*TN*): 120 (patients truly negative).
- False positive (*FP*): 68 (patients negative but classified as positive).
- False negative (*FN*): 73 (patients positive but classified as negative).

**Figure 8:**
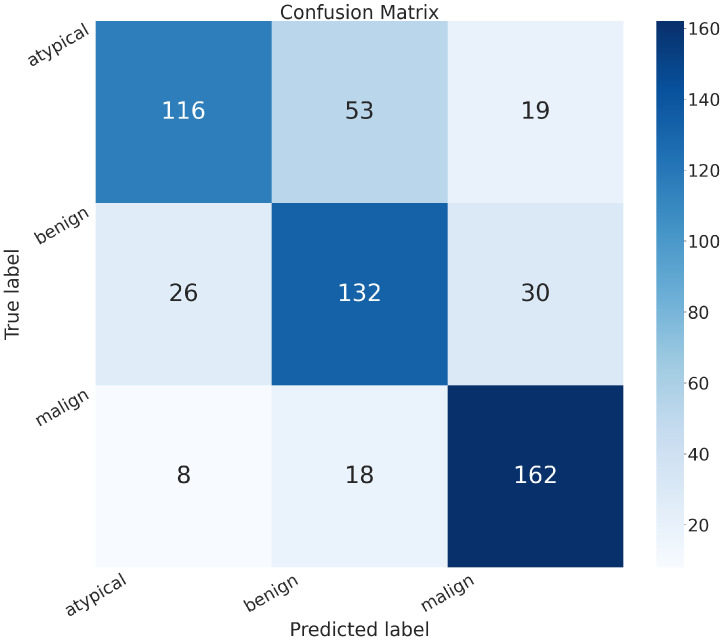
Confusion matrix obtained with a fine-tuning of the MobileNet model.

The confusion matrix for the MobileNet model with fine-tuning, in Figure 8, shows the following:

- True positive (*TP*): 305 (patients truly positive), with 135 affected by atypical 170 and by malignant tumors.
- True negative (*TN*): 132 (patients truly negative).
- False positive (*FP*): 56 (patients negative but classified as positive).
- False negative (*FN*): 71 (patients positive but classified as negative).

Comparing it with the confusion matrix of the MobileNet model without fine-tuning, a slight worsening was noted, especially in the number of true positives.

**Figure 9:**
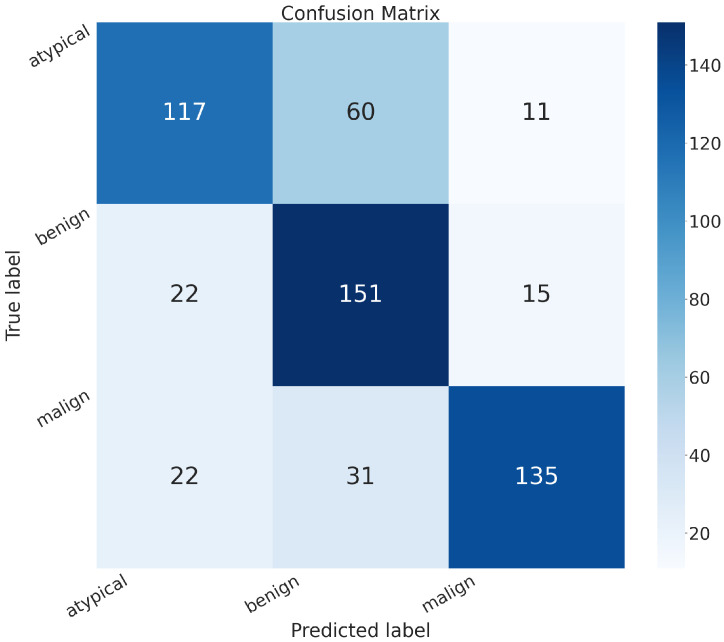
Confusion matrix obtained with a fine-tuning of the VGG-19 model.

The confusion matrix of the VGG-19 model with fine-tuning, in Figure 9, shows the following:

- True positive (*TP*): 285 (patients truly positive), with 128 affected by atypical 157 and by malignant tumors.
- True negative (*TN*): 151 (patients truly negative).
- False positive (*FP*): 37 (patients negative but classified as positive).
- False negative (*FN*): 91 (patients positive but classified as negative).

In this case, there was a lower number of true positives, true negative, and false positive compared to the confusion matrix without fine-tuning.

Comparing all of these confusion matrices, the MobileNet and VGG-19 models without fine-tuning still appeared superior to the other methods. They both exhibited higher values along the main diagonal, which means that they had a lower rate of misclassification than all of the other methods analyzed.

### 3.2. Qualitative Analysis

Drawing our conclusion merely on metrics would lead us to consider the network as a black box, while we wanted to propose a method that could be explainable to boost adoption of deep learning in real-world medical activity. For this purpose, we also referred to the images obtained through Grad-CAM, a technique that has proved to be extremely valuable for Explainable Artificial Intelligence (XAI). The generated images present in fact a heatmap that visually highlights the areas the model relied on to make its decisions. That way, we can understand more thoroughly the reasons behind the classification carried out by a machine learning model. A heatmap conveys information through a color scale; specifically, in the images shown below, significant regions are represented in yellow, while less important areas exhibit a blue/violet color. We can also see some green areas while transitioning from a relevant one to an overlooked one. With regard to the qualitative analysis, the results were discussed with domain experts who validated the output of the explainability.

**Figure 10:**
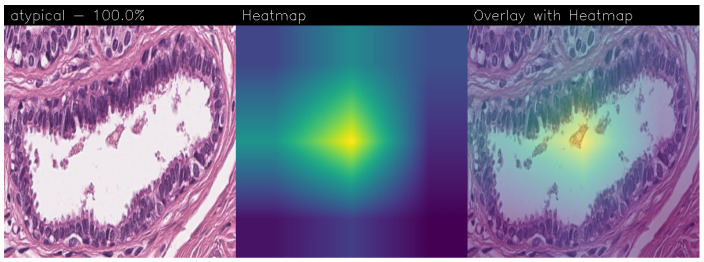

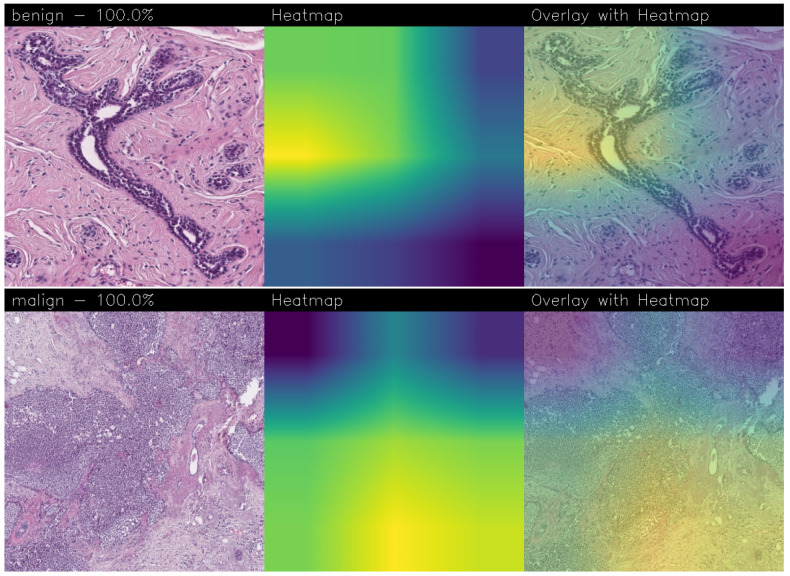
Heatmaps obtained from the MobileNet.

**Figure 11:**
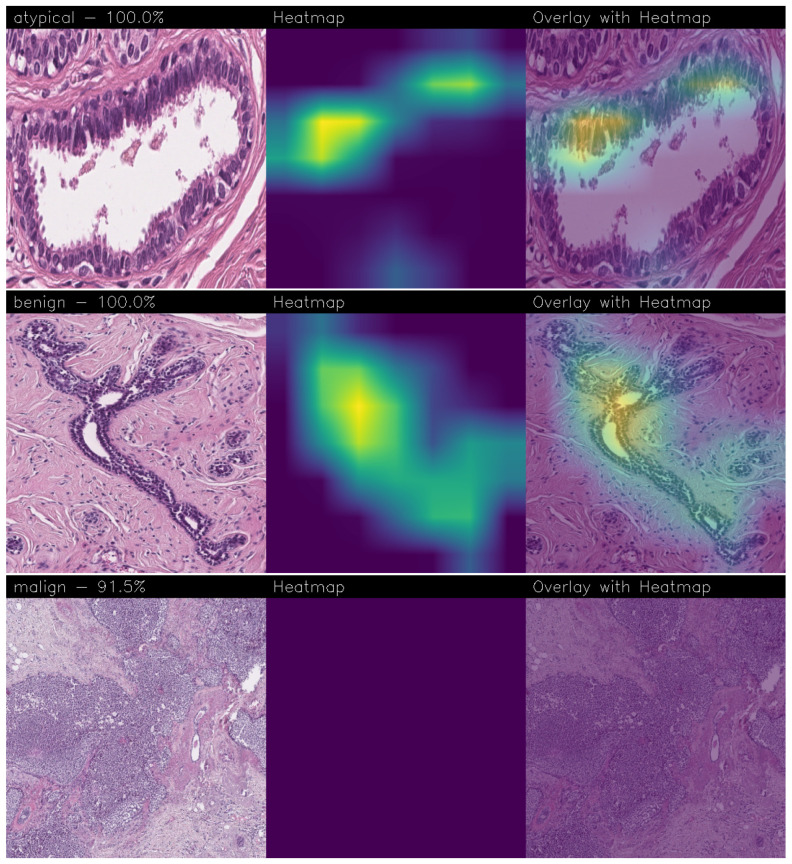
Heatmaps obtained from the VGG-19 model.

Below are the Grad-CAM heatmaps obtained from the MobileNet model (Figure 10):

Below are the Grad-CAM heatmaps obtained from the VGG-19 model (Figure 11):

Despite the high accuracy achieved, the MobileNet and VGG-19 models without fine-tuning show Grad-CAM heatmaps with really poor precision. Looking at the heatmaps obtained, we notice how in both cases, they highlight large areas of the image, with an extremely high level of confidence (100%) but without any particular reason. The only exception found is for the malignant case for the VGG-19 model, which shows a confidence of 91.5%, but the heatmap is completely empty, everything is violet. It means that the network correctly decided to classify the image as malignant, but it was practically a random process.

**Figure 12:**
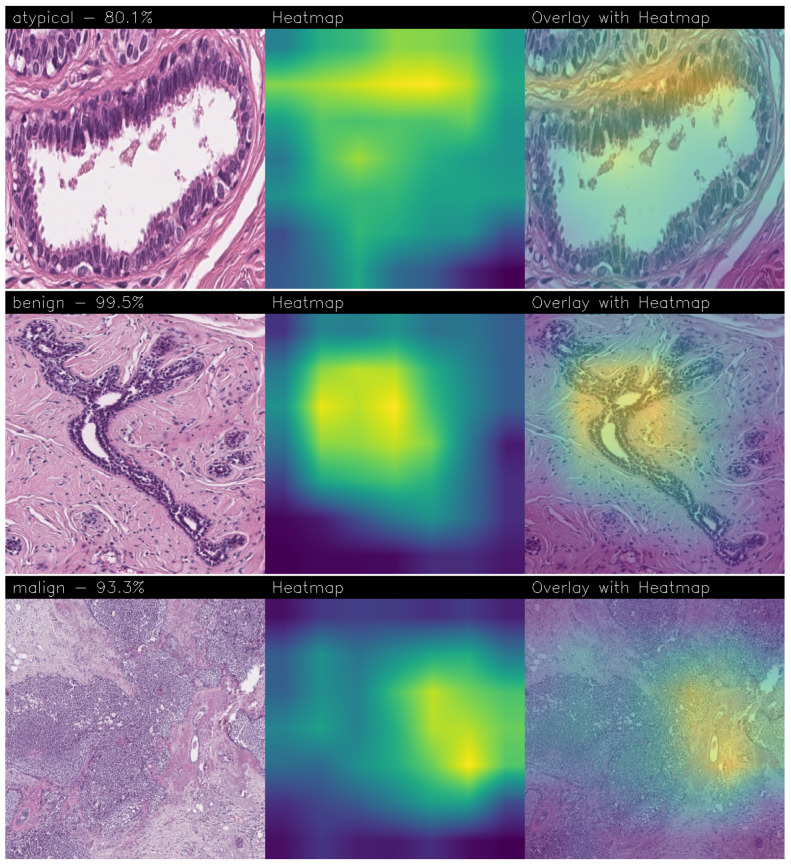
Heatmaps obtained from the MobileNet model with fine-tuning.

**Figure 13:**
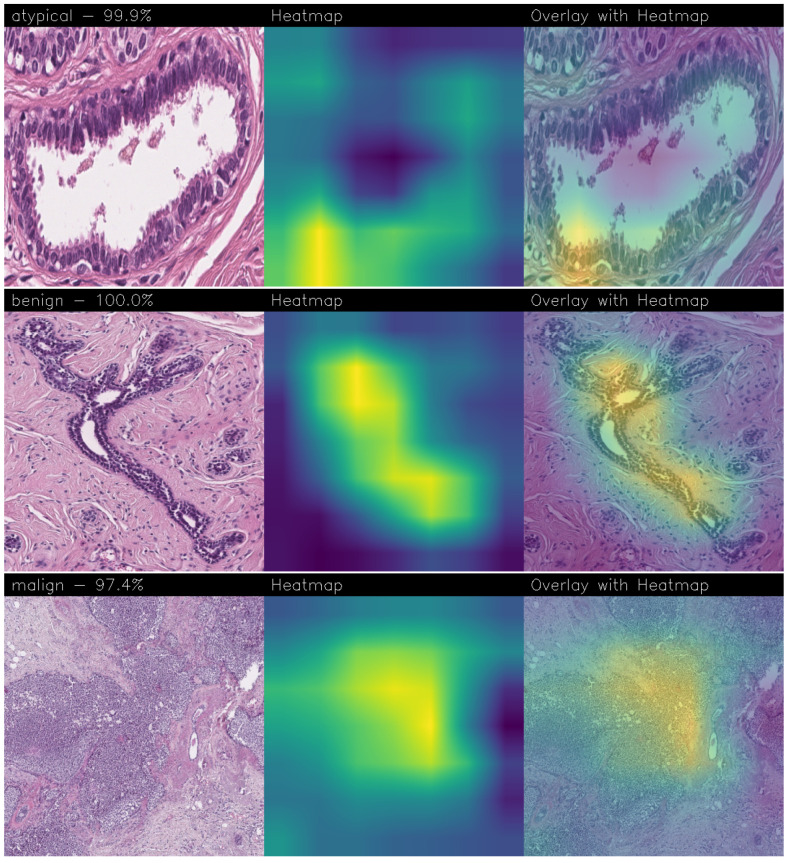
Heatmaps obtained from the VGG-19 model with fine-tuning.

Below are the Grad-CAM heatmaps obtained from the MobileNet model with fine-tuning (Figure 12):

Below are the Grad-CAMs obtained from the VGG-19 model with fine-tuning (Figure 13):

We stated in the previous Section 3.1 that fine-tuning degraded the performance of the models, mostly the level of accuracy. This was also detected in the level of confidence associated with the images, which appeared to be lower. However, it was also clear that their capacity for recognizing the signs of the disease improved since the significant regions were smaller and presented more evident edges. Looking at the overlay of the heatmap with the images, it also seemed that the edges defined an area that was close to the actual region of interest, thus validating the classification.

**Figure 14:**
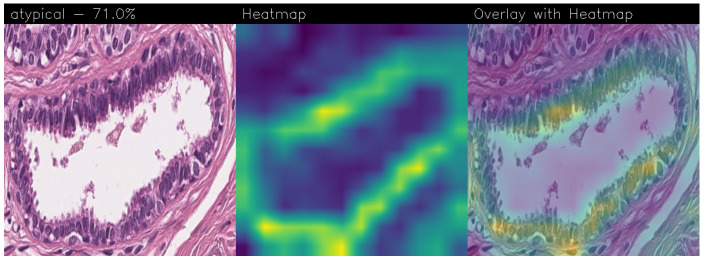
Heatmaps related to atypical cancer, classified as 71.0% obtained with the Standard_CNN model.

**Figure 15:**
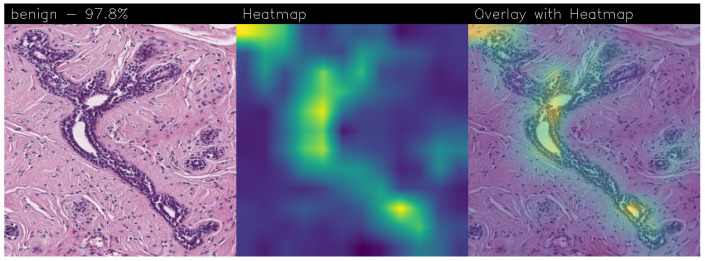
Heatmaps related to benign cancer, classified as 97.8% obtained with the Standard_CNN model.

**Figure 16:**
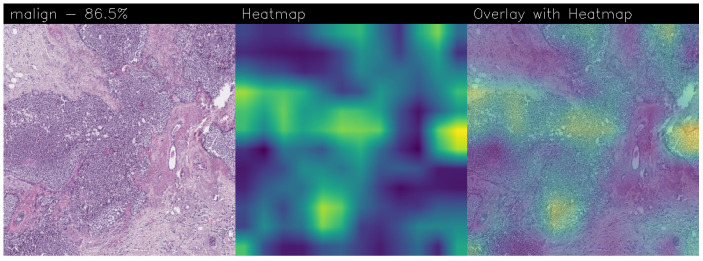
Heatmaps related to malign cancer, classified as 86.5% obtained with the Standard_CNN model.

**Figure 17:**
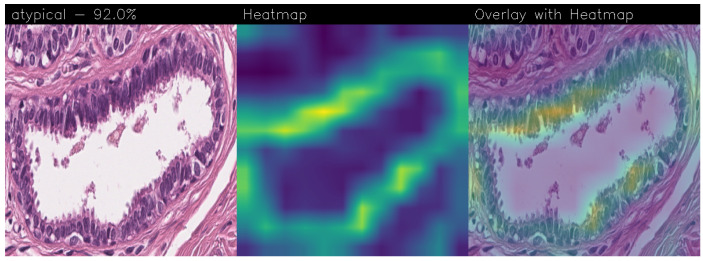
Heatmaps related to atypical cancer, classified as 92.0% obtained with the MR_Net model.

**Figure 18:**
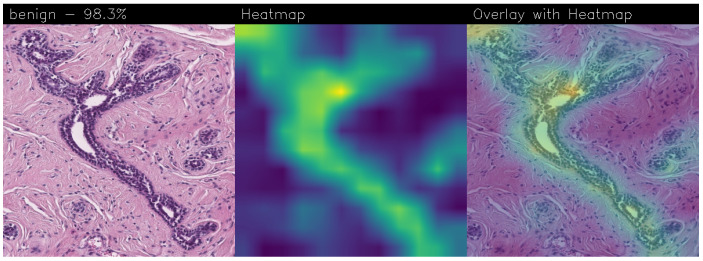
Heatmaps related to benign cancer, classified as 98.3% obtained with the MR_Net model.

**Figure 19:**
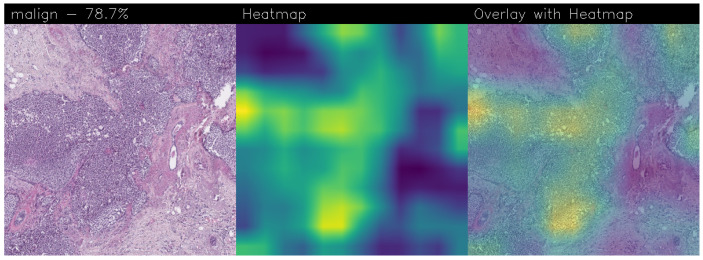
Heatmaps related to malign cancer, classified as 78.7% obtained with the MR_Net model.

Below are the Grad-CAM heatmaps obtained from the Standard_CNN model (Figure 14):

Below are the Grad-CAM heatmaps obtained from the MR_Net model:

Analyzing the Grad-CAMs obtained with both Standard_CNN (Figure 14, Figure 15 and Figure 16) and MR_Net (Figure 17, Figure 18 and Figure 19), we can clearly see the improvement over the previous models. They actually provided precise edges for relevant areas, which almost perfectly overlapped with the region in the histological image that showed signs of the disease. The differences between these two models were subtle; however, with a careful analysis, it was evident that the results of the MR_Net model were closer to reality.

This was confirmed by also conducting an analysis that focused more on the histopathological aspect of the images:

- In the case of the atypical category (a precancerous condition of the breast), the classifier utilized the area where the breast duct walls were a darker purple in color. Those walls were a little too thick with an excessive number of cells, since epithelial atypia can grow to a thickness of five or six cubic epithelial cells, as opposed to the normal thickness of the breast duct lining of about two cells. In fact, epithelial atypia is a proliferation of epithelial cells in the terminal duct–lobular units (TDLU) of the breast. The cells are clustered in acini that have rigid contours, round nuclei, and even chromatin, and the cell borders are readily appreciated, creating the impression of a mosaic pattern. Secretions and calcifications are present in the acinar lumens.
- In the case of the benign category, the classifier detected the area of normal tissue, consisting of glandular tissue and adipose tissue. Ducts, lobules, and acini of the mammary gland are lined with epithelial cells and immersed in adipose tissue. The model focused on areas of the image containing the fibroadenoma, a benign pathological nodule that results from the proliferation of the glandular epithelium and fibrous stroma of the breast. It is characterized by a fibroblastic stroma with glandular structures with cystic spaces, surrounded by connective tissue forming an enveloping capsule.
- In the case of the malignant category, the classifier relied on large areas of the image, characterized by undifferentiated malignant tissue, in which the tumor cells had lost all their specific, normal histological features and were therefore difficult to classify. In fact, it was an invasive carcinoma.

### 3.3. Comparison of Heatmaps: MR_Net vs. Standard_CNN

In this subsection, we conduct an in-depth analysis of the heatmaps generated by the MR_Net and Standard_CNN models, i.e., the models that emerged from experimental analysis with the ability to localize BC. As demonstrated in this section, the difference in accuracy between the MR_Net and Standard_CNN models was approximately 1%, beyond the increase in neurons, but from the explainability point of view, the two models exhibited a different behavior. As a matter of fact, the explainability of a model is critical in the medical field, especially for tasks like BC detection, where understanding the basis of a model’s prediction can build trust among clinicians and support informed decision-making. Heatmaps, specifically those generated using Grad-CAM, were used to visualize the regions within histological images that the model focused on when making a prediction.

#### 3.3.1. Heatmap Analysis of MR_Net Model

The MR_Net model’s heatmaps provided precise and focused visualizations of the regions that contributed most to its decisions. In the case of histological images classified as malignant, MR_Net highlighted the areas with a high cellular density and irregular structures, characteristic of invasive carcinomas. The generated heatmaps showed concentrated attention on specific clusters of cells, highlighting the morphological abnormalities such as cell shape irregularities and tissue architecture disorganization. This precision allowed pathologists to understand which specific parts of an image led the model to classify it as malignant.

For images classified as atypical, the MR_Net model demonstrated a strong focus on areas of ductal hyperplasia, where the breast duct walls appear thickened. These regions are typically characterized by a proliferation of epithelial cells that exceeds normal cell thickness. The heatmaps generated for atypical cases effectively outlined the areas where the cell structure deviated from normality, thereby aiding in the identification of early-stage precancerous lesions. Such detailed attention enhanced the interpretability of the model’s predictions, making MR_Net a more transparent tool for medical diagnosis.

#### 3.3.2. Heatmap Analysis of Standard_CNN Model

In contrast, the heatmaps produced by the Standard_CNN model displayed less precise localization of significant regions within the images. For malignant cases, the attention appeared to be more dispersed across the image, often highlighting regions that did not contain relevant pathological features. The model’s focus was spread over larger areas, including those that might not directly indicate the presence of invasive carcinoma. This broader attention reduced the model’s ability to pinpoint specific cancerous regions, making it more challenging for clinicians to trust the prediction outcomes.

For images classified as atypical, the Standard_CNN model showed a similar lack of specificity in its heatmaps. The attention was often directed towards larger portions of the tissue that did not necessarily correspond to the regions showing cellular abnormalities. As a result, the model’s decisions appeared less justifiable to domain experts, since the heatmaps did not provide clear visual explanations for the classifications made.

#### 3.3.3. Comparison and Implications for Explainability

The most interesting difference between the MR_Net and Standard_CNN models lies in the clarity and focus of their heatmaps. As a matter of fact, the MR_Net model’s ability to concentrate on pathologically relevant regions allows it to offer a more interpretable decision-making process. For instance, when identifying malignant tissues, MR_Net consistently highlighted areas with increased cellular proliferation and disorganized structures, which are key indicators of invasive carcinoma. This aligns closely with how pathologists manually analyze histological images, suggesting that the MR_Net model’s predictions are based on clinically significant features.

In contrast, the broader focus areas of the Standard_CNN model’s heatmaps may lead to confusion or misinterpretation. The lack of precise localization could result in predictions that are more difficult to justify, reducing the confidence of pathologists in relying on the model’s output for clinical decisions. Additionally, the dispersed attention could indicate that the Standard_CNN model is learning features that are not necessarily relevant to the classification task, leading to a higher potential for errors in its predictions.

#### 3.3.4. Remarks on Explainability

In summary, the MR_Net model demonstrated better prediction explainability when compared to the Standard_CNN model, as evidenced by both qualitative evaluations of the generated heatmaps. Its focused attention on clinically relevant areas allowed it to provide more interpretable predictions, making it a valuable tool for aiding pathologists in diagnosing breast cancer. The Standard_CNN model, while effective in certain scenarios, lacked the precision required for high-stakes medical applications where understanding the basis of a decision is essential. These findings underscore the importance of integrating explainability into the development of deep learning models for medical imaging.

## 4. Related Work

This section provides a comprehensive analysis of BC classification by discussing the most significant studies. This review aims to assist researchers in the field of BC classification by offering a clear and concise overview of existing challenges, solutions, and potential future directions.

One notable study on BC classification through deep learning was conducted by Brancati, Nadia, et al. [21], in which the authors introduced the BReAst Carcinoma Subtyping (BRACS) dataset. This dataset is a large collection of annotated hematoxylin and eosin (HE)-stained images designed to advance AI development in the automatic characterization of breast lesions. BRACS contains 547 whole-slide images (WSIs) and 4539 regions of interest (ROIs) extracted from the WSIs. Each WSI and its corresponding ROIs were annotated into different lesion categories by the consensus of three board-certified pathologists. Specifically, BRACS includes three lesion types—benign, malignant, and atypical—further subtyped into seven categories. To the best of our knowledge, it is the largest annotated dataset for breast cancer subtyping at both the WSI and ROI levels. Moreover, by incorporating the often-understudied atypical lesions, BRACS provides a unique opportunity to leverage AI to gain a deeper understanding of their characteristics.

Another significant study was from Ahmed, Fahad, et al. [22], where the BRACS dataset of histological HE-stained images was used to classify breast cancer tumors. That dataset includes both whole-slide images (WSI) and region-of-interest (ROI) images, though the authors focused on ROI images for their study. Various pre-trained deep learning models, such as Xception, EfficientNet, ResNet50, and InceptionResNet, were experimented with, using ImageNet pre-trained weights. The BRACS ROI images were preprocessed with techniques such as image augmentation, upsampling, and specific dataset split strategies. For the default dataset split, ResNet50 achieved the best results with a 66% F1-score. For the custom dataset split, the best results were achieved through upsampling and image augmentation, resulting in a 96.2% F1-score. This approach also significantly reduced false positive and false negative classifications to less than 3% for each class. The study primarily focused on identifying seven breast cancer tumor subtypes.

A third study by Kausar, Tasleem, Yun Lu, and Adeeba Kausar [23] involved experiments conducted on three publicly available benchmark histopathology image datasets. The proposed model demonstrated multiclass classification accuracy of 96.25%, 99.8%, and 72.2% on the International Conference on Image Analysis and Recognition (ICIAR 2018), BreakHis, and BRACS datasets, respectively. The reported inference time per image for the proposed model was 0.67 s for ICIAR 2018 and 0.21 s for BreakHis and BRACS images. This study aimed to address the computational cost issue of deep automatic systems. Instead of directly using the input images, the wavelet transform (WT) was applied to decompose the images into different frequency bands, with only the low-frequency bands subjected to the proposed lightweight deep convolutional neural network (CNN). The lightweight deep model was designed using an invertible residual block module. Incorporating the invertible residual block module into the deep CNN model, along with the use of the WT, significantly reduced the computational cost of the proposed model without a noticeable decline in accuracy. Additionally, the impact of various machine vision classifiers—such as support vector machine (SVM), softmax, and K-nearest neighbors classifier (KNN)—on model performance was analyzed.

Thus, it is emerging that the application of deep learning to BC detection has garnered significant attention in recent years. This section reviews various approaches and models aimed at enhancing the accuracy of BC detection from histological images. The focus is on methods that leverage CNNs and their performance on different datasets.

In this context, Brancati et al. [21] introduced the BReAst Carcinoma Subtyping (BRACS) dataset, which includes 547 whole-slide images (WSIs) and 4539 regions of interest (ROIs). Using pre-trained CNNs like ResNet and EfficientNet, the study aimed to classify seven breast lesion types, achieving 66% accuracy on the ROI dataset. The inclusion of atypical lesions made the dataset unique and valuable for training models to distinguish between various subtypes of breast cancer.

Ahmed et al. [22] applied transfer learning using models like Xception, EfficientNet, and ResNet50 on the BRACS ROI dataset. Their study focused on fine-tuning these models using ImageNet pre-trained weights. Through advanced data augmentation and custom dataset splitting, they achieved an F1-score of 96.2%, significantly improving classification performance and reducing false positives and false negatives to less than 3% per class.

Kausar et al. [23] proposed a lightweight CNN architecture for multiclass classification on three datasets, including BRACS. The method used wavelet transforms for feature extraction, reducing computational cost while maintaining high accuracy. The study reported 72.2% accuracy on BRACS, 96.25% on the ICIAR 2018 dataset, and 99.8% on the BreakHis dataset.

Nawaz et al. [7] used AlexNet for classifying breast cancer images from the ICIAR 2018 dataset, achieving 87% accuracy. The study explored the application of transfer learning with AlexNet, fine-tuning it on histology images to improve detection of carcinoma subtypes.

Sahu et al. [4] developed a hybrid CNN for breast cancer classification using mammogram and ultrasound images. Their model combined features from both imaging modalities to achieve 94.5% accuracy, demonstrating the effectiveness of multi-modal deep learning approaches for improving diagnostic accuracy.

He et al. [12] introduced a fusion-based multi-output method for stroke risk classification and applied a similar approach for breast cancer detection. The study focused on combining multiple CNN models to enhance specificity and sensitivity, resulting in improved detection rates for atypical lesions in histological images.

Tan and Le [17] developed the EfficientNet architecture, which scaled model dimensions uniformly for improved performance. Applied to breast histology images, EfficientNet-B0 achieved high efficiency and accuracy, outperforming other standard models like ResNet and VGG on the BRACS dataset.

Simonyan and Zisserman [19] proposed VGG-16 and VGG-19, which are now widely used in medical image classification tasks, including breast cancer detection. When fine-tuned on the BRACS dataset, these models reached up to 73% accuracy, showing their potential in capturing intricate details in histological images.

Howard et al. [20] introduced MobileNet, a lightweight network that was suitable for mobile and embedded vision applications. When applied to the BRACS dataset, MobileNet provided a balance between accuracy and inference speed, achieving a classification accuracy of 73% while being computationally efficient.

He et al. [18] proposed ResNet-50, which has become a standard for deep learning tasks. It was applied to breast cancer detection on the BRACS dataset, achieving an accuracy of 71.6% by leveraging residual connections to improve gradient flow during training.

Gurcan et al. [9] reviewed the application of histopathological image analysis for cancer detection, highlighting the transition from traditional methods to AI-based techniques. The review emphasized the potential of CNNs in automating diagnosis and reducing pathologist workload.

Di Giammarco et al. [13] proposed a robust deep learning method for cervical cancer screening, adapted for breast cancer studies. The method focused on interpretability through Grad-CAM visualizations, providing insights into the model’s decision-making process for histopathological images.

Vaidyanathan and Kaklamani [6] examined the role of genetic factors in breast cancer, and how CNNs could be utilized to integrate imaging data with genetic information for more accurate predictions. This approach aimed to combine radiological data with genomics for personalized diagnostics.

Table 5 shows a comparison of the state-of-the-art literature related to BC detection.

As emerged from the discussion, this work represents the first attempt to propose a method for explainable BC detection and localization.

## 5. Conclusions and Future Work

Accurate histopathological diagnosis is crucial for BC as patient numbers surge and pathologist resources dwindle [24]. We believe that our study significantly impacts the early diagnosis and identification of breast cancer tumors and their subtypes, especially atypical and malignant tumors, thus improving patient outcomes and reducing patient mortality rates. Although the proposed model did not outperform state-of-the-art models in terms of BC detection, it did in terms of explainability, as the heatmaps generated using Grad-CAM revealed a proper detection of the presence of benign, atypical, and malignant tumors. Both our networks (Standard_CNN and MR_Net) based their decision on the geometry of the structures and the number and shape of the cells. However, MR_NET managed to obtain more defined contours for the areas of interest, despite presenting a slightly lower level of confidence. Neither already existing models nor the fine-tuned ones seemed to reach these results for the Grad-CAM heatmaps. It is clear that these models did not evaluate the correct areas of the images, thus partially invalidating their results. Integrating deep learning into routine pathology practice stands to improve diagnostic accuracy, thereby contributing to reducing avoidable errors. As future work, we will explore the possibility of considering other models, for instance, related to object detection, to understand whether it is possible to improve the performance obtained in terms of BC localization. Moreover, we are working to provide numerical values of the coincidence of the regions obtained by Grad-CAM with the image areas for decision-making, with the aim to compute a kind of metric for the evaluation of the qualitative analysis.

## Figures and Tables

**Table 1:** The hyperparameters selected during the experimentation.

Model	Image Size	Batch	Epochs	Learning Rate	Ex. Time
Standard CNN	110 × 100 × 3	32	20	0.0001	0:08:57
EfficientNet	224 × 224 × 3	32	20	0.00001	1:21:48
ResNet50	110 × 110 × 3	32	50	0.0001	2:24:58
VGG-16	224 × 224 × 3	32	50	0.00001	15:44:49
VGG-19	224 × 224 × 3	32	50	0.00001	18:40:30
MobileNet	110 × 110 × 3	32	20	0.001	0:13:08
MR_Net	110 × 110 × 3	32	20	0.0001	0:18:08

**Table 2:** First-phase experimental analysis results.

Model	Accuracy	Loss	Precision	Recall
EfficientNet	0.6738	0.9003	0.6875	0.6631
ResNet50	0.7163	1.5485	0.7163	0.7163
VGG-16	0.7269	1.3160	0.7337	0.7181
VGG-19	0.7305	1.4156	0.7338	0.7234
MobileNet	0.7305	1.5697	0.7351	0.7234
Standard CNN	0.6737	0.9034	0.6824	0.6401

**Table 3:** Second-phase experimental analysis results.

Model	Accuracy	Loss	Precision	Recall
MR_Net	0.6897	0.8354	0.7028	0.6542

**Table 4:** Fine-tuning experimental analysis results.

Model	Accuracy	Loss	Precision	Recall
MobileNet	0.7270	0.7132	0.7589	0.7198
VGG-16	0.7234	0.8434	0.7431	0.7181
VGG-19	0.7145	1.1417	0.7171	0.7057

**Table 5:** Comparison of the current state-of-the-art literature on BC detection.

Study	Task	Model	Dataset	Performance
Brancati et al. (2022) [21]	Subtyping BC lesions	ResNet, EfficientNet	BRACS	66% accuracy
Ahmed et al. (2023) [22]	Transfer learning	Xception, ResNet50	BRACS	96.2% F1-score
Kausar et al. (2023) [23]	Multiclass BC classification	Lightweight CNN	BRACS	72.2% accuracy
Nawaz et al. (2018) [7]	BC classification	AlexNet	ICIAR 2018	87% accuracy
Sahu et al. (2023) [4]	Hybrid model for BC	Hybrid CNN	Mammogram + Ultrasound	94.5% accuracy
He et al. (2024) [12]	Fusion for BC detection	Multi-CNN Fusion	Custom	Improved specificity
Tan and Le (2019) [17]	Model scaling for BC	EfficientNet-B0	BRACS	High efficiency
Simonyan and Zisserman (2014) [19]	Fine-tuned CNNs	VGG-16, VGG-19	BRACS	73% accuracy
Howard et al. (2017) [20]	Mobile vision	MobileNet	BRACS	73% accuracy
He et al. (2016) [18]	Deep residual learning	ResNet-50	BRACS	71.6% accuracy
Gurcan et al. (2009) [9]	Review of AI in histology	Traditional + AI methods	Various	Review study
Di Giammarco et al. (2023) [13]	Explainable cancer detection	Custom CNN	Cervical images	High interpretability
Vaidyanathan and Kaklamani (2021) [6]	Genetic data integration	Custom CNN	Genetic + Imaging	Improved precision
Nam et al. (2020) [24]	AI in pathology	Various CNNs	Digital pathology	Integration challenges

## Data Availability

The dataset is freely available for research purposes at the following url: https://www.bracs.icar.cnr.it/.
